# Bi-stability and period-doubling cascade of frequency combs in exceptional-point lasers

**DOI:** 10.1515/nanoph-2025-0069

**Published:** 2025-05-22

**Authors:** Xingwei Gao, Hao He, Weng W. Chow, Alexander Cerjan, Chia Wei Hsu

**Affiliations:** Ming Hsieh Department of Electrical and Computer Engineering, 5116 University of Southern California, Los Angeles, CA, 90089, USA; Center for Integrated Nanotechnologies, Sandia National Laboratories, Albuquerque, NM, 87185, USA

**Keywords:** frequency combs, exceptional points, lasers

## Abstract

Recent studies have demonstrated that a laser can self-generate frequency combs when tuned near an exceptional point (EP), where two cavity modes coalesce. These EP combs induce periodic modulation of the population inversion in the gain medium, and their repetition rate is independent of the laser cavity’s free spectral range. In this work, we perform a stability analysis that reveals two notable properties of EP combs, bi-stability and a period-doubling cascade. The period-doubling cascade enables halving of the repetition rate while maintaining the comb’s total bandwidth, presenting opportunities for the design of highly compact frequency comb generators.

## 1 Introduction

A frequency comb is an optical phenomenon where a system produces a series of equally-spaced spectral lines. Optical frequency combs (OFCs) are essential to optical-frequency synthesizers [1] and precision metrology [2], [3], [4]. In the past decade, OFCs have also been applied in optical communications [5] and quantum computation [6]. Conventionally, OFCs are generated by mode locked lasers [7], optical cavities with nonlinearity [8], [9], [10], and quantum cascade lasers [11], [12], [13]. However, these traditional comb generation methods all require the repetition rate to match the free spectral range (FSR) of the laser cavity. Consequently, large cavity sizes are required to generate radio-frequency OFCs.

Recently, it was discovered that exceptional point (EP) optical cavities can develop into frequency combs with repetition rates independent of the cavity’s FSR [14], [15], [16]. EPs are points of degeneracy in the phase space where two or more eigenmodes become identical [17], [18], [19], [20], [21]. When two cavity modes are sufficiently close to an EP, there exists a gain threshold above which any perturbation to the system induces a long-lived periodic modulation to the carrier populations in the gain medium. These dynamic populations subsequently modulate any active lasing modes in the system, generating equally spaced comb lines in the output spectrum. As such, an EP comb is self-generated without any external modulation, and its repetition rate *ω*_d_, equal to the self-modulation rate of the inversion, can be significantly smaller than the cavity’s free spectral range as it is approximately set by the frequency spacing of the EP modes. In principal, arbitrarily small repetition rates can be achieved as the laser system is tuned sufficiently close to the EP. However, in practice, it is technically difficult to reach an exact EP in experiments. Moreover, the robustness of the comb will be compromised due to the enhanced sensitivity near EP [16], [21], [22]. Therefore, the minimum achievable repetition rate in EP combs is thought to be limited.

In this work, we show that one can significantly reduce the repetition rate of EP combs without pushing the system closer to the EP, thus realizing robust radio-frequency OFCs in small laser cavities. Specifically, we demonstrate that an EP comb can halve its repetition rate multiple times through a period-doubling cascade, which was typically observed in more complicated laser systems with light injection or an external modulation [23], [24]. In particular, we carry out a stability analysis with the perturbation method on the Maxwell-Bloch equations [25], [26]. Driven by the periodic population inversion, an EP laser becomes a Floquet system [27], where any infinitesimal perturbation can be decomposed into Floquet eigenmodes, with each having a complex Floquet frequency. A Floquet frequency with a positive (negative) imaginary part leads to the corresponding Floquet mode growing (decaying) over time. The stability of an EP comb is thus determined by the sign of Im(*ω*_F_), with *ω*_F_ being the Floquet frequency with the largest imaginary part. Given an existing EP comb with a line spacing of *ω*_d_, by solving for *ω*_F_, we find a series of pumping thresholds at which a Floquet mode turns on, with Im(*ω*_F_) = 0 and Re(*ω*_F_) = 0.5*ω*_d_. Through gain saturation, this Floquet mode induces an additional modulation to the population inversion, which has twice the period of the inversion fluctuation from the original EP comb. The re-modulated inversion then doubles the period of the lasing field’s envelope, hence inserting extra lines into the original EP comb. The period doubling occurs through each of these thresholds cascade, eventually leading to arbitrarily small repetition rate. With the perturbation method, we also find a bistability zone in EP lasers, where two different EP combs exist at the same pumping strength. Thus, the laser state depends on initialization, which is potentially applicable in optical signal-processing devices and all-optical computer systems [28].

## 2 Stability analysis on frequency combs

Period doubling is a special transition from one frequency comb to another. For the transition to occur, the former comb must first become unstable. To predict the stability of a comb, we apply the first-order perturbation method to the fundamental equations governing lasing materials. We begin by deriving generalized perturbation equations for arbitrary lasing states. Then, we extend the analysis to limit-cycle lasing states, proving that the stability of a frequency comb is associated with a complex Floquet frequency *ω*_F_, which can be determined by solving a linear eigenmode equation.

Lasers can be described rigorously by the Maxwell–Bloch (MB) equations [16], [25], [26], a semi-classical model depicting the relations among the population inversion *D*(**r**, *t*) of gain media, the electric field **E**(**r**, *t*) and the polarization density **P**(**r**, *t*). To simplify the notation, we focus on a one-dimensional (1D) laser cavity with $E\left(r,t\right)=E\left(x,t\right)\hat{z}$ , $P\left(r,t\right)=P\left(x,t\right)\hat{z}$ and *D*(**r**, *t*) = *D*(*x*, *t*) (Our method can be readily extended to three-dimensional systems, as shown in Supplementary section I). In particular, the MB equations are

(1) $$\frac{\partial }{\partial t}D=-\gamma_{\|}\left(D-D_{\text{p}}\right)-\frac{i\gamma_{\|}}{2}\left(E^{*}P-EP^{*}\right),$$

(2) $$\frac{\partial }{\partial t}P=-\left(i\omega_{ba}+\gamma_{⊥}\right)P-i\gamma_{⊥}DE,$$

(3) $$\frac{\partial^{2}E}{\partial x^{2}}-\frac{1}{c^{2}}\left(\varepsilon_{c}\frac{\partial^{2}}{\partial t^{2}}+\frac{\sigma }{\varepsilon_{0}}\frac{\partial }{\partial t}\right)E=\frac{1}{c^{2}}\frac{\partial^{2}}{\partial t^{2}}P.$$

*D*, *E*, and *P* here have been normalized by *R* ^2^/(*ɛ*_0_ *ℏγ*_⊥_), $2R/\left(\hbar \sqrt{\gamma_{⊥}\gamma_{\|}}\right)$ , and $2R/\left(\varepsilon_{0}\hbar \sqrt{\gamma_{⊥}\gamma_{\|}}\right)$ , respectively, with *R* being the amplitude of the atomic dipole moment, *ɛ*_0_ the vacuum permittivity, *ℏ* the Planck constant, *γ*_‖_ the population relaxation rate and *γ*_⊥_ the dephasing rate of the gain-induced polarization (*i.e.*, the bandwidth of the gain). *D*_p_(*r*) is the normalized net pumping strength and profile, *ω*_*ba*_ is the frequency gap between the two atomic levels, *ɛ*_*c*_(*x*) is the relative permittivity profile of the cold cavity, *σ*(*x*) is a conductivity profile that produces linear absorption, and *c* is the vacuum speed of light.

Consider a fixed-point or limit-cycle solution to the MB equations (1)–(3), *D* = *D*_s_(*x*, *t*), *E* = *E*_s_(*x*, *t*) and *P* = *P*_s_(*x*, *t*). To determine the stability of the solution, we add a small perturbation, such that *D* = *D*(*x*,*t*)_s_ + Δ*d*(*x*, *t*), *E* = *E*_s_(*x*, *t*) + Δ*ϵ*(*x*, *t*), and *P* = *P*_s_(*x*, *t*) + Δ*p*(*x*, *t*), where Δ is a real infinitesimal number. The perturbation equations are derived by substituting the perturbed *D*, *P* and *E* into Eqs. (1)–(3) and then extracting the linear terms of Δ,

(4) $$\frac{\partial }{\partial t}d=-\gamma_{\|}d-\frac{i\gamma_{\|}}{2}\left(\left\{E_{\text{s}}^{*}p+P_{\text{s}}\epsilon^{*}\right\}-\text{c.c.}\right),$$

(5) $$\frac{\partial }{\partial t}p=-\left(i\omega_{ba}+\gamma_{⊥}\right)p-i\gamma_{⊥}\left(D_{s}\epsilon +E_{s}d\right),$$

(6) $$\frac{\partial^{2}\epsilon }{\partial x^{2}}-\frac{1}{c^{2}}\left(\varepsilon_{c}\frac{\partial^{2}}{\partial t^{2}}+\frac{\sigma }{\varepsilon_{0}}\frac{\partial }{\partial t}\right)\epsilon =\frac{1}{c^{2}}\frac{\partial^{2}}{\partial t^{2}}p.$$

Notably, for small *D*_p_, the laser is off, hence *E*_s_ = 0, *P*_s_ = 0 and *D*_s_ = *D*_p_. At such a trivial state, equations (4)–(6) yield linear-cavity wave equations [29],

(7) $$\frac{\partial^{2}\epsilon_{m}}{\partial x^{2}}+\frac{{\tilde{\omega }}_{m}^{2}}{c^{2}}\left(\varepsilon_{c}+\frac{i\sigma }{\varepsilon_{0}{\tilde{\omega }}_{m}}+\Gamma_{⊥}\left({\tilde{\omega }}_{m}\right)D_{\text{p}}\right)\epsilon_{m}=0,$$

where $\Gamma_{⊥}\left(\omega \right)=\frac{\gamma_{⊥}}{\omega -\omega_{ba}+i\gamma_{⊥}}$ . Equation (7) determines the linear cavity’s resonant modes *ϵ*_*m*_ and the related resonant frequencies ${\tilde{\omega }}_{m}$ . An EP is approached when two resonant modes merge.

Above the first lasing threshold, a single mode turns on. The solution is a non-trivial fixed point, $E_{\text{s}}=E_{0}\left(x\right){\text{e}}^{-\text{i}\omega_{0}t}$ , $P_{\text{s}}=P_{0}\left(x\right){\text{e}}^{-\text{i}\omega_{0}t}$ and *D*_s_ = *D*_0_(*x*). Fixed-point stability analysis has been applied to the study of the sensitivity and signal-to-noise ratio of nonlinear EP sensors [30]. Under the stationary-inversion approximation (SIA), Eqs. (4)–(6) yield active-cavity modes and determine the second lasing threshold [29].

Beyond the SIA, Eqs. (4)–(6) determine the comb threshold at which single-mode lasing becomes unstable and the system transitions to a limit cycle [16],

(8) $$E_{\text{s}}\left(x,t\right)={\text{e}}^{-\text{i}\omega_{0}t}\overset{+\infty }{\underset{m=-\infty }{\sum }}E_{m}\left(x\right)e^{-im\omega_{\text{d}}t},$$

(9) $$P_{\text{s}}\left(x,t\right)={\text{e}}^{-\text{i}\omega_{0}t}\overset{+\infty }{\underset{m=-\infty }{\sum }}P_{m}\left(x\right)e^{-im\omega_{\text{d}}t},$$

(10) $$D_{\text{s}}\left(x,t\right)=\overset{+\infty }{\underset{m=-\infty }{\sum }}D_{m}\left(x\right)e^{-im\omega_{\text{d}}t},$$

where the repetition rate *ω*_d_, spectral center *ω*_0_ and the Fourier components {*D*_*m*_, *E*_*m*_, *P*_*m*_} can all be determined by “periodic-inversion *ab initio* laser theory” (PALT) [16]. Eqs. (8)–(10) act as a periodic temporal modulation in Eqs. (4)–(6). By Floquet theory [27], the solution to Eqs. (4)–(6) should include Floquet modes, $f_{k}\left(x,t\right){\text{e}}^{-\text{i}\omega_{\text{F},k}t}$ , where *f*_*k*_(*x*, *t*) has a period of 2*π*/*ω*_d_. Due to the difference-frequency generation terms involving ϵ* and p* in Eq. (4), each harmonic oscillation ${\text{e}}^{-\text{i}\omega_{\text{F},k}t}$ generates a complex-conjugate term ${\text{e}}^{\text{i}\omega_{\text{F},k}^{*}t}$ . Hence, we postulate the following form of solutions,

(11) $$\epsilon \left(x,t\right)={\text{e}}^{-\text{i}\omega_{0}t}\underset{k}{\sum }\left[\epsilon_{ak}{\text{e}}^{-\text{i}\omega_{\text{F},k}t}+\epsilon_{bk}^{*}{\text{e}}^{\text{i}\omega_{\text{F},k}^{*}t}\right],$$

(12) $$p\left(x,t\right)={\text{e}}^{-\text{i}\omega_{0}t}\underset{k}{\sum }\left[p_{ak}{\text{e}}^{-\text{i}\omega_{\text{F},k}t}+p_{bk}^{*}{\text{e}}^{\text{i}\omega_{\text{F},k}^{*}t}\right],$$

(13) $$d\left(x,t\right)=\underset{k}{\sum }d_{ak}{\text{e}}^{-\text{i}\omega_{\text{F},k}t}+d_{ak}^{*}{\text{e}}^{\text{i}\omega_{\text{F},k}^{*}t}.$$

Here, *f*_*ak*,*bk*_ with *f* = *ϵ*, *p*, *d* are functions of *x* and *t*, with a time period of 2*π*/*ω*_d_, *f*_*ak*,*bk*_ = *f*_*ak*,*bk*_(*x*, *t*) = *f*_*ak*,*bk*_(*x*, *t* + 2*π*/*ω*_d_). To determine *ω*_F_ and the space-time profile of *ϵ*_*a*,*b*_(*x*, *t*), we substitute Eqs. (8)–(10) and Eqs. (11)–(13) into Eqs. (4)–(6). By expanding the periodic functions in Fourier series, we derive the following wave equations,

(14) $$\frac{d^{2}}{dx^{2}}\left[\begin{array}{c} {\bar{\epsilon }}_{ak}\\ {\bar{\epsilon }}_{bk} \end{array}\right]+\Omega \left(\omega_{\text{F},k}\right)\left[\begin{array}{c} {\bar{\epsilon }}_{ak}\\ {\bar{\epsilon }}_{bk} \end{array}\right]=X\left(x,\omega_{\text{F},k}\right)\left[\begin{array}{c} {\bar{\epsilon }}_{ak}\\ {\bar{\epsilon }}_{bk} \end{array}\right],$$

in which ${\bar{\epsilon }}_{ak,bk}\left(x\right)$ is a column vector that includes the Fourier components of *ϵ*_*ak*,*bk*_(*x*, *t*). **Ω**(*ω*_F,*k*_) and **X**(*ω*_F,*k*_) are matrices determined by the limit cycle, {*E*_s_, *P*_s_, *D*_s_}. (*d* ^2^/d*x* ^2^) acts element-wise on the column vector. $\left(d^{2}{\bar{\epsilon }}_{ak}/dx^{2}\right)$ isssss associated with outgoing boundaries while $\left(d^{2}{\bar{\epsilon }}_{bk}/dx^{2}\right)$ is associated with incoming boundaries. The derivation of Eq. (14) and the expressions for **Ω**(*ω*_F,*k*_) and **X**(*x*, *ω*_F,*k*_) are provided in the supplementary section I.

Floquet modes can be solved from Eq. (14). Their Floquet frequencies *ω*_F,*k*_ are determined such that the eigenvectors ${\bar{\epsilon }}_{ak,bk}\left(x\right)$ are non-trivial. For each *ω*_F,*k*_, the expressions of Eq. (11)–(13) suggest that $\omega_{\text{F},k^{\prime }}\equiv -\omega_{\text{F},k}^{*}$ and *ω*_F,*k*_″ ≡ *ω*_F,*k*_ + *mω*_d_ with m∈Z are degenerate solutions to the same Floquet mode. Therefore, all the Floquet frequencies can be mapped into the “Floquet zone”, defined by Re(*ω*) ∈ [0, 0.5*ω*_*d*_] on the complex plane. In the Floquet zone, we define the primary Floquet frequency *ω*_F_ as the *ω*_F,*k*_ with the largest imaginary part, then denote the related mode profile in the expression of Eq. (11) as ϵ_*a*,*b*_(*x*, *t*). A comb is stable if and only if all perturbations decay over time, which requires Im(*ω*_F,*k*_) < 0 for all *k*, equivalently Im(*ω*_F_) < 0.

## 3 Bistability and period doubling cascade of EP combs

**Figure 1:**
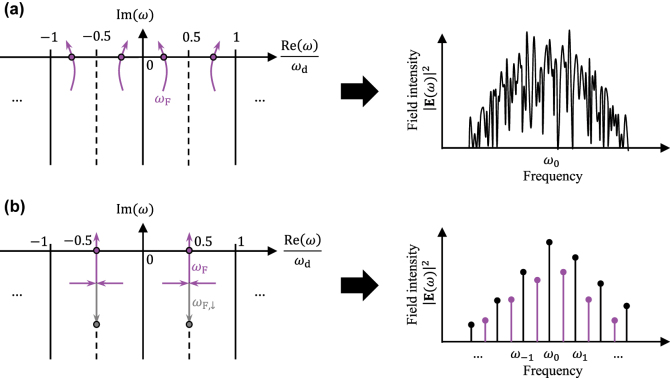
The Floquet frequencies *ω*_F_ and the spectra resulting from *ω*_F_ > 0. The original comb lines are located at *ω*_*m*_ = *ω*_0_ + *mω*_d_ for *m* = 0, ±1, …, with *ω*_0_ being the comb center and *ω*_d_ the repetition rate. In the left column, pink arrows are the Floquet frequency *ω*_F_ and its partner eigenvalues in other zones, namely $\left(-\omega_{\text{F}}^{*}+k\omega_{\text{d}}\right)$ and (*ω*_F_ + *kω*_d_), evolving with the pumping strength. (a) *ω*_F_ crosses the real axis at an irrational ratio to *ω*_d_, resulting in a randomly fluctuating spectrum. (b) *ω*_F_ reaches the zone boundary (0.5*ω*_d_) simultaneously with $\left(-\omega_{\text{F}}^{*}+\omega_{\text{d}}\right)$ below the real axis, then splits into two non-degenerated solutions, which we label as *ω*_F_ and *ω*_F,__*↓*_, respectively. *ω*_F_ crosses the real axis along the zone boundary, while *ω*_F,__*↓*_ is repelled down. Such behavior leads to another comb with half the repetition rate as the original comb.

While the imaginary part of the primary Floquet frequency *ω*_F_ generally determines the stability of a frequency comb, the real part implies how the comb evolves after it becomes unstable. Specifically, period doubling occurs when (i) Im(*ω*_F_) = 0 and (ii) Re(*ω*_F_) = 0.5*ω*_d_. Under condition (i), a random perturbation reduces to a single Floquet mode over time, $\epsilon \left(x,t\right)\to {\text{e}}^{-\text{i}\left(\omega_{0}+\omega_{\text{F}}\right)t}g\left(x,t\right)$ , where $g\left(x,t\right)=\left(\epsilon_{a}+\epsilon_{b}^{*}e^{2i\omega_{\text{F}}t}\right)$ . A stronger pumping will lead to the nonlinear effect of wave mixing between the growing Floquet mode and the original comb. If Re(*ω*_F_)/*ω*_d_ is irrational, such wave mixing process will generate an overcomplicated lasing spectrum spreading over all frequencies, illustrated in Figure 1a. However, condition (ii) indicates that *g*(*x*, *t* + 2*π*/*ω*_d_) = *g*(*x*, *t*), hence *ϵ*(*x*, *t*) consists of Fourier components at *ω*_0_ + (*m* + 0.5)*ω*_d_, right in the middle of the existing comb lines. Therefore, wave mixing only generates frequencies of *ω*_0_ + 0.5*mω*_*d*_, forming another frequency comb with half the repetition rate as before, as shown by Figure 1b. The fact that *ω*_F_ reaches exactly 0.5*ω*_d_ in condition (ii) is not a coincidence. As discussed in Section 2, both *ω*_F_ and $-\omega_{\text{F}}^{*}+\omega_{\text{d}}$ are eigenvalues of Eq. (14). These two frequencies are necessarily symmetric about the Floquet zone boundary at 0.5*ω*_d_, and are therefore constrained to appear as a pair. Consequently, when *ω*_F_ reaches the zone boundary, it must collide with its partner. As the pumping strength increases further, this degeneracy splits in one of two possible ways: horizontally or vertically. In the case of horizontal splitting, the two solutions acquire different real parts while maintaining the same imaginary part. They remain paired and generically move away from the zone boundary, yielding irregular dynamics if they reach the real axis as illustrated in Figure 1a. In contrast, vertical splitting keeps both solutions pinned to the zone boundary with equal real parts but different imaginary parts, such that each solution becomes its own partner. We denote these vertically separated solutions as *ω*_F_ and *ω*_F,*↓*_ in Figure 1b, where *ω*_F_ is the one that eventually crosses the real axis. Any deviation from this behavior – for example, if one branch were to leave the zone boundary – would require the appearance of additional solutions such as $-\omega_{\text{F}}^{*}+\omega_{\text{d}}$ or $-\omega_{\text{F},↓}^{*}+\omega_{\text{d}}$ , violating the conservation of eigenvalue count *ω*_F,*k*_ as defined in Eq. (14).

**Figure 2:**
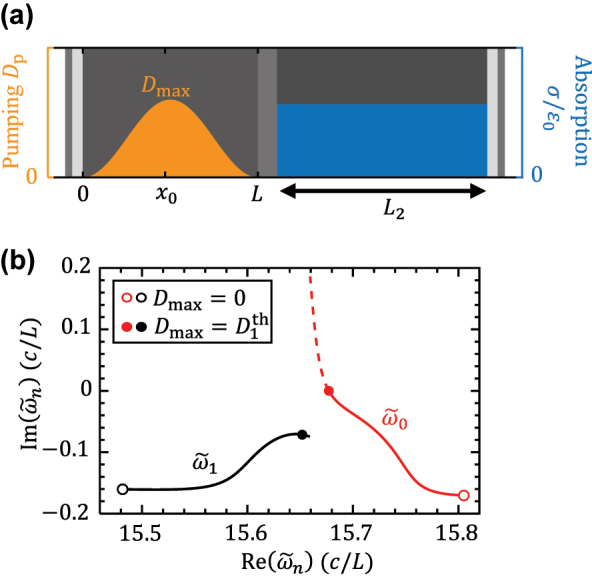
The EP laser with gain-loss coupled cavity. (a) A 1D laser cavity with gain on the left and loss on the right. The length of the gain cavity is *L* and the length of the loss cavity is *L*_2_ = 1.2*L*. The absorption rate inside the loss cavity is *σ*/*ɛ*_0_ = 7.6(*c*/*L*). The passive refractive index $\sqrt{\varepsilon_{c}}$ is 3.4 in the gain cavity and 3.67 in the passive cavity, typical values of commonly used semiconductor lasing materials. *x*_0_ is the location where we plot the field intensity in Figure 3 and the phase portraits in Figure 5. The values of all passive parameters, including the thickness and the index of each layer, are elaborated in supplementary section II. (b) the trajectories of two near-EP resonant frequencies solved from Eq. (7). The first lasing threshold is $D_{1}^{\text{th}}=1.2$ . Dashed lines show the would-be above-threshold trajectories in the absence of gain saturation.

We demonstrate such period doubling mechanism in a one-dimensional EP laser cavity shown in Figure 2a. We adopt a smooth pumping profile *D*_p_(*x*) = 0.5*D*_max_[1 − cos(2*πx*/*L*)] to improve the accuracy of the finite-difference time-domain (FDTD) simulations. Figure 2b shows the trajectories of two eigen frequencies solved from Eq. (7); they are tuned near an EP at the first threshold $D_{\max}=D_{1}^{\text{th}}$ , where ${\tilde{\omega }}_{0}$ reaches the real axis, as shown in Figure 2b. Without nonlinear gain saturation, ${\tilde{\omega }}_{0}$ would move quickly upward as *D*_max_ increases above $D_{1}^{\text{th}}$ , while ${\tilde{\omega }}_{1}$ would almost stay still, due to the counteraction between being pumped and being repelled by ${\tilde{\omega }}_{0}$ .

**Figure 3:**
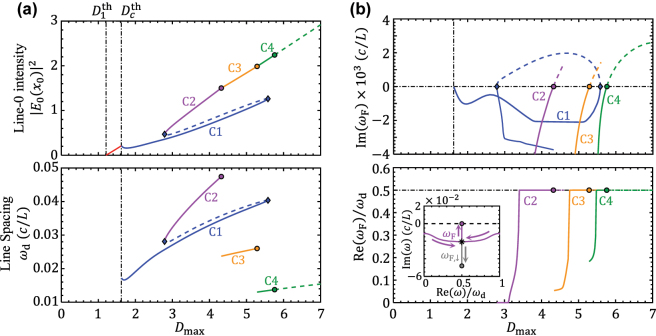
Pump dependence of EP combs. (a) PALT calculation of EP combs. (Upper panel) The intensity of central comb line and (lower panel) the repetition rate of EP combs. (Solid lines) Stable solutions and (dashed lines) unstable solutions. (Blue diamonds) the boundaries of bistability. (circles) Period doubling, where *ω*_d_ drops by half. (b) The imaginary part (upper panel) and real part (lower panel) of primary Floquet frequencies *ω*_F_ solved from Eq. (14) for the combs in a. The inset is the trajectory of C2’s Floquet frequency *ω*_F_ in the complex plane, where arrows indicate the increasing of *D*_max_. The result corresponds the case of vertical splitting illustrated in Figure 1b.

For this system, the frequency gap $|\text{Re}\left({\tilde{\omega }}_{1}-{\tilde{\omega }}_{0}\right)|=0.03\left(c/L\right)$ at the first threshold is an estimation to the repetition rate of the EP comb near the comb threshold. It is approximately 30 times smaller than the gain cavity’s FSR, $\omega_{\text{FSR}}=\pi c/\sqrt{\varepsilon_{c}}≃0.9\left(c/L\right)$ . Without tuning the system closer to the EP, we now show how the repetition can be significantly reduced through period-doubling cascade. Figure 3a shows the PALT calculation of the laser states depending on the pumping strength. The upper panel shows a continuous transition from the single lasing mode $\left(D_{1}^{\text{th}}<D_{\max}\leq D_{c}^{\text{th}}\right)$ to the major comb line *E*_0_(*x*_0_) across the comb threshold $D_{c}^{\text{th}}$ . The lower panel shows the corresponding repetition rate of the EP combs above $D_{c}^{\text{th}}$ . Different comb solutions are labeled as “C”-branches with different colors. Figure 3b shows the solutions of primary Floquet frequencies *ω*_F_ for the EP combs in Figure 3a. Stable combs are associated with Im(*ω*_F_) < 0. After an *ω*_F_ crosses the real axis, the corresponding comb transits from one branch to another.

**Figure 4:**
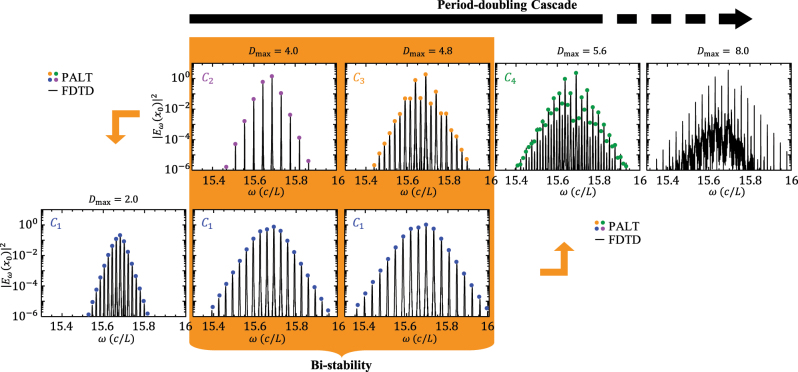
PALT and FDTD simulation of EP-comb spectra at different pumping strengths. Upper row shows the period-doubling cascade. The bi-stable region is highlighted in orange. Orange arrows show two different paths of how an EP comb evolves. When the pumping *D*_max_ increases regularly from 2.0 to 8.0, the simulation converges to the spectra on the lower row for *D*_max_ = 4.0 and 4.8, then jumps to *C*4 spectrum on the upper row. When the pumping *D*_max_ reduces regularly from 8.0 to 2.0, the simulation converges to the upper row for *D*_max_ = 4.0 and 4.8, then jumps down to *C*1.

The filled circles in Figure 3a mark the thresholds of period doubling, corresponding to the points where *ω*_F_ = 0.5*ω*_d_ in Figure 3b. Above each of these thresholds, the repetition rate reduces by half, shown in the lower panel of Figure 3a. Figure 4 shows the PALT calculation and FDTD simulation of the lasing spectra at several different pumping strengths selected from Figure 3. The extra comb lines induced by the Floquet mode can be identified on the comb spectrum at *D*_max_ = 4.8, compared to the spectrum at *D*_max_ = 4.0. The two spectra also show similar comb bandwidth, implying that the emergence of new comb lines does not compromise the intensity of the existing lines.

As the number of comb lines increases, solving PALT and Eq. (14) becomes increasingly time-consuming due to the large number of Fourier components required to maintain accuracy. As a result, although both PALT and the Floquet-theorem-based stability analysis are theoretically sound, they become impractical beyond *C*4. In this regime, FDTD simulations reveal a continuous spectrum at *D*_max_ = 8.0, which can arise from one of two possible evolution paths: either an infinite cascade of period doublings or a random crossing of the real axis by *ω*_F_, as illustrated in Figure 1a. Additional simulation results provided in the Supplementary Section III demonstrate two more period doublings (i.e., *C*5–*C*6). Beyond this point, the repetition rate becomes too small to resolve within the finite simulation times considered here. A future theoretical framework is needed to ultimately distinguish between these two evolution paths.

In addition to period doubling cascade, our stability analysis also predicts bistable EP combs within the pumping range of 2.8 < *D*_max_ < 5.6. In this region, both *C*1 and the *C*2–*C*3–*C*4 chain are stable. The two stable branches are accessed by different initial conditions. The accessibility is demonstrated by FDTD simulations in Figure 4. First, we set the pumping strength to be $D_{\max}=2.0>D_{\text{c}}^{\text{th}}$ and initialize the simulation with a random pulse of the electrical field inside the gain cavity. After the lasing state converges onto the limit cycle, we increase the pumping strength by a small step, and then continue the simulation. We iterate such simulation process until *D*_max_ = 8.0. The simulated comb stays on *C*1 branch (lower row of the spectra in Figure 4), then suddenly jumps up onto *C*4 (indicated by the right arrow) as *D*_max_ crosses the right end of *C*1 at 5.6. Second, we start the simulation at *D*_max_ = 8.0, then regularly reduce the pumping strength. The simulated comb changes backwardly along *C*4 → *C*3 → *C*2, then suddenly jumps down onto *C*1 as *D*_max_ crosses the left end of *C*2 at 2.8 (indicated by the left arrow). Thus, the simulation results demonstrate both PALT and the stability analysis on EP combs.

**Figure 5:**
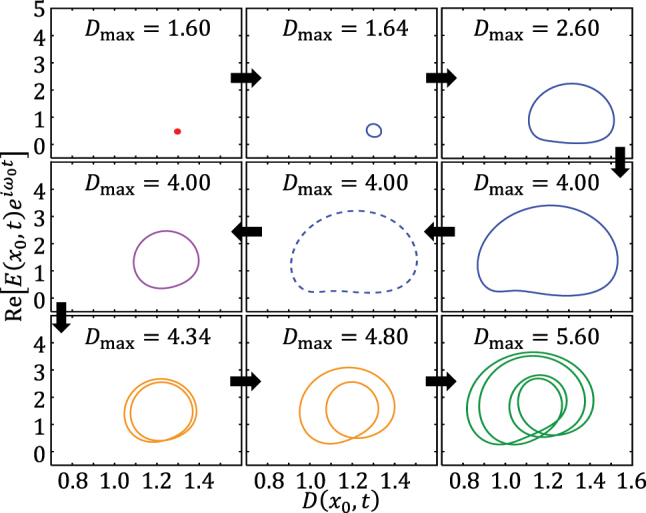
The projection of phase portraits on the plane of population inversion versus electrical field at ***x* = *x***_**0**_. The horizontal axis is the population inversion *D*_*s*_(*x*_0_, *t*) and vertical axis is the envelope of the electrical field *E*_*s*_. Line styles and colors are consistent with Figure 3.

Finally, we summarize the period doubling and bistability phenomena using the phase trajectories of EP combs. The laser’s phase space is defined as a manifold with dimensions of *D* as well as the real and imaginary parts of *E* and *P* from Maxwell–Bloch equations Eqs. (1)–(3). A frequency comb is then recognized as a limit cycle in the phase space [31]. For the solutions in Figures 3a and 5 plots the projections of their phase portraits on the *D*(*x*_0_, *t*)–Re[*E*(*x*_0_, *t*)] plane. The red dot in the upper left plot is the single-mode lasing state, known as a fixed point. The first row shows how the fixed point opens and continuously extends into a stable limit cycle. The second row shows the limit cycle shifting from an attractor (right) to a repeller (middle), then reverting to an attractor (left) again. This gives rise to bi-stability. The third row shows period-doubling cascade, where the limit cycle with a basic period doubles its orbit twice.

## 4 Conclusions

In this work, we develop a limit-cycle stability analysis based on Floquet theory. The analysis predicts the novel phenomena of bi-stability and period-doubling cascade of frequency combs in EP lasers. Period doubling cascade occurs when the Floquet frequency crosses real axis at half of the comb’s repetition rate. It reduces the repetition rate without shrinking the comb bandwidth, hence allowing for the design of extremely compact OFC generators. The theoretical results are confirmed by FDTD simulations. The four-wave mixing phenomenon observed in a laser diode coupled to a high-Q resonator [32] may represent an experimental demonstration of such period-doubling cascade in EP combs. This system possesses all the essential ingredients of an EP: two modes with similar frequencies (one from the laser diode and the other from the high-Q micro-ring cavity), field coupling via backscattering, and gain-loss compensation (gain from the lasing material and loss from the passive cavity).

Our perturbation analysis can be extended to solve scattering problems of periodically-driven nonlinear systems. By including a term of incident wave in Eq. (11), one can re-derive Eq. (14) with an extra inhomogeneous source term. The Flouqet frequency will then be recognized as *ω*_in_ − *ω*_0_, where *ω*_in_ is the frequency of the incident wave. Future work can study the scattering spectrum of EP lasers by solving such inhomogeneous perturbation equation.

## Supplementary Material

Supplementary Material Details
