# Supplementary material for: Bi-stability and period-doubling cascade of frequency combs in exceptional-point lasers
Source: Nanophotonics. 2025 May 22;14(13):2259–66. doi: 10.1515/nanoph-2025-0069 (PMC12199559; doi:10.1515/nanoph-2025-0069)
Supplement: Supplementary file 1 — Supplementary Material Details [file j_nanoph-2025-0069_suppl_001.pdf]

# Supplementary Information for Bi-stability and Period-doubling Cascade of Frequency Combs in Exceptional-point lasers

## 1. DERIVATION OF STABILITY EQUATIONS

Here, we derive the equation governing comb stability, as presented in Eq. (14) of the main text, by solving the perturbation equations, Eqs. (4)–(6). In the process, we provide explicit expressions for the entries of the coefficient matrices, $\mathbf{X}(\mathbf{r}, \omega_{\mathrm{F},k})$ and $\Omega(\omega_{\mathrm{F},k})$. To ensure the generality of the method, the derivation is conducted in three dimensions (3D), after which the result is simplified to obtain Eq. (14) for one dimensional systems.

The 3D Maxwell–Bloch(MB) equations for electrical field $\mathbf{E}(\mathbf{r}, t)$, polarization $\mathbf{P}(\mathbf{r}, t)$ and population inversion $D(\mathbf{r}, t)$ are

$$\frac{\partial}{\partial t} D = -\gamma_{\parallel}(D - D_{\mathrm{p}}) - \frac{i\gamma_{\parallel}}{2}(\mathbf{E}^* \cdot \mathbf{P} - \mathbf{E} \cdot \mathbf{P}^*), \tag{S1}$$

$$\frac{\partial}{\partial t}\mathbf{P} = -(i\omega_{ba} + \gamma_{\perp})\mathbf{P} - i\gamma_{\perp} D(\mathbf{E} \cdot \hat{\theta})\hat{\theta}^*, \tag{S2}$$

$$-\nabla \times \nabla \times \mathbf{E} - \frac{1}{c^2}\left(\varepsilon_c \frac{\partial^2}{\partial t^2} + \frac{\sigma}{\varepsilon_0}\frac{\partial}{\partial t}\right)\mathbf{E} = \frac{1}{c^2}\frac{\partial^2}{\partial t^2}\mathbf{P}. \tag{S3}$$

$\hat{\theta}$ is the unit vector of the atomic dipole momentum, with $\hat{\theta} \cdot \hat{\theta}^* = 1$. The rest of the notation is consistent with Eq. (1)–(3) in the main text. Similar to Eqs. (4)–(6), the 3D perturbation equations can be derived as:

$$\frac{\partial}{\partial t} d = -\gamma_{\parallel} d - \frac{i\gamma_{\parallel}}{2}(\{\mathbf{E}_{\mathrm{s}}^* \cdot \mathbf{p} + \mathbf{P}_{\mathrm{s}} \cdot \boldsymbol{\epsilon}^*\} - \mathrm{c.c.}), \tag{S4}$$

$$\frac{\partial}{\partial t}\mathbf{p} = -(i\omega_{ba} + \gamma_{\perp})\mathbf{p} - i\gamma_{\perp}[(D_{\mathrm{s}}\boldsymbol{\epsilon} + \mathbf{E}_{\mathrm{s}} d) \cdot \hat{\theta}]\hat{\theta}^*, \tag{S5}$$

$$-\nabla \times \nabla \times \boldsymbol{\epsilon} - \frac{1}{c^2}\left(\varepsilon_c \frac{\partial^2}{\partial t^2} + \frac{\sigma}{\varepsilon_0}\frac{\partial}{\partial t}\right)\boldsymbol{\epsilon} = \frac{1}{c^2}\frac{\partial^2}{\partial t^2}\mathbf{p}, \tag{S6}$$

where $\mathbf{E}_{\mathrm{s}}$, $\mathbf{P}_{\mathrm{s}}$ and $D_{\mathrm{s}}$ solves the Maxwell–Bloch(MB) equations (1)–(3) in the main text. We consider a known limit-cycle solution,

$$\mathbf{E}_{\mathrm{s}}(\mathbf{r}, t) = e^{-i\omega_0 t}\mathbf{E}_{\mathrm{env}}(\mathbf{r}, t) = e^{-i\omega_0 t}\sum_{m=-\infty}^{+\infty}\mathbf{E}_m(\mathbf{r})e^{-im\omega_{\mathrm{d}} t}, \tag{S7}$$

$$\mathbf{P}_{\mathrm{s}}(\mathbf{r}, t) = e^{-i\omega_0 t}P_{\mathrm{env}}(\mathbf{r}, t)\hat{\theta}^* = e^{-i\omega_0 t}\sum_{m=-\infty}^{+\infty}P_m(\mathbf{r})e^{-im\omega_{\mathrm{d}} t}\hat{\theta}^*, \tag{S8}$$

$$D_{\mathrm{s}}(\mathbf{r}, t) = \sum_{m=-\infty}^{+\infty} D_m(\mathbf{r})e^{-im\omega_{\mathrm{d}} t}. \tag{S9}$$

where the Fourier components, $\{\mathbf{E}_m, P_m, D_m\}$, repetition rates, $\omega_{\mathrm{d}}$ and spectral center $\omega_0$ have been determined by "periodic-inversion *ab initio* laser theory"(PALT).[1] In this situation, Eq. (S4)–(S6) describes a Floquet system under periodic modulation. Therefore, the solutions of $[d, p, \epsilon]$ should be enveloped by Floquet states. Noting the complex–conjugate operation (c.c.) in Eq. (S4), we consider the trial solution as

$$\boldsymbol{\epsilon}(\mathbf{r}, t) = e^{-i\omega_0 t}\sum_k[\boldsymbol{\epsilon}_{ak}(\mathbf{r}, t)e^{-i\omega_{\mathrm{F},k} t} + \boldsymbol{\epsilon}_{bk}^*(\mathbf{r}, t)e^{i\omega_{\mathrm{F},k}^* t}], \tag{S10}$$

$$\mathbf{p}(\mathbf{r}, t) = e^{-i\omega_0 t}\sum_k[p_{ak}(\mathbf{r}, t)e^{-i\omega_{\mathrm{F},k} t} + p_{bk}^*(\mathbf{r}, t)e^{i\omega_{\mathrm{F},k}^* t}]\hat{\theta}^*, \tag{S11}$$

$$d(\mathbf{r}, t) = \sum_k[d_{ak}(\mathbf{r}, t)e^{-i\omega_{\mathrm{F},k} t} + d_{ak}^*(\mathbf{r}, t)e^{i\omega_{\mathrm{F},k}^* t}]. \tag{S12}$$

$\boldsymbol{\epsilon}_{a,b}$, $p_{a,b}$ and $d_{ak}$ have the same temporal period of $2\pi/\omega_{\rm d}$. The form of Eq.(S12) ensures that $d$ is real, while $\boldsymbol{\epsilon}^*_{bk}$ or $p^*_{bk}$ cannot be assumed as the complex conjugate of $\boldsymbol{\epsilon}_{ak}$ or $p_{ak}$, meaning $\boldsymbol{\epsilon}_{bk} \neq \boldsymbol{\epsilon}_{ak}$ and $p_{bk} \neq p_{ak}$ in general. Now, the target is to determine these periodic envelopes as well as their complex Floquet frequencies $\omega_{{\rm F},k}$. First, we substitute Eq.(S7)–(S8) and Eq.(S10)–(S12) into Eq.(S4) then extract the terms of $e^{-i\omega_{{\rm F},k}t}$ for each $k$. To simplify the notation, we now omit the index $k$, as the following derivation applies to all Floquet modes, including the one with the primary Floquet frequency $\omega_{\rm F}$.

$$(\frac{\partial}{\partial t} - i\omega_{\rm F} + \gamma_{\|})d_a = -\frac{i\gamma_{\|}}{2}(E^*_{\rm env}p_a + P_{\rm env}\epsilon_b - E_{\rm env}p_b - P^*_{\rm env}\epsilon_a), \tag{S13}$$

where $E_{\rm env} = \mathbf{E}_{\rm env}\cdot\hat{\theta}$, $\epsilon_b = \boldsymbol{\epsilon}_b\cdot\hat{\theta}^*$ and $\epsilon_a = (\boldsymbol{\epsilon}_a\cdot\hat{\theta})$. The $e^{i\omega_{\rm F}t}$ component is simply the complex conjugate of Eq. (S13). Then, we substitute Eq.(S7), (S9) and (S10)–(S12) into Eq.(S5), then separate the term of $e^{-i(\omega_0-\omega^*_{\rm F})t}$ from that of $e^{-i(\omega_0+\omega_{\rm F})t}$. The term of $e^{-i(\omega_0+\omega_{\rm F})t}$ yields

$$[\frac{\partial}{\partial t} - i(\omega_0 + \omega_{\rm F} - \omega_{ba}) + \gamma_\perp]p_a = -i\gamma_\perp(D_{\rm s}\epsilon_a + E_{\rm env}d_a). \tag{S14}$$

The complex conjugate of $e^{-i(\omega_0-\omega^*_{\rm F})t}$ term yields

$$[\frac{\partial}{\partial t} + i(\omega_0 - \omega_{\rm F} - \omega_{ba}) + \gamma_\perp]p_b = i\gamma_\perp(D_{\rm s}\epsilon_b + E^*_{\rm env}d_a), \tag{S15}$$

where we have applied the fact that population inversion is real, $D^*_{\rm s} = D_{\rm s}$. Similarly, we substitute Eq. (S10)–(S11) into the wave equation Eq. (S6), which yields

$$-\nabla\times\nabla\times\boldsymbol{\epsilon}_a - \frac{1}{c^2}\left[\varepsilon_c(\frac{\partial}{\partial t} - i\omega_0 - i\omega_{\rm F})^2 + \frac{\sigma}{\varepsilon_0}(\frac{\partial}{\partial t} - i\omega_0 - i\omega_{\rm F})\right]\boldsymbol{\epsilon}_a = \frac{1}{c^2}(\frac{\partial}{\partial t} - i\omega_0 - i\omega_{\rm F})^2 p_a\hat{\theta}^*, \tag{S16}$$

$$-\nabla\times\nabla\times\boldsymbol{\epsilon}^*_b - \frac{1}{c^2}\left[\varepsilon_c(\frac{\partial}{\partial t} - i\omega_0 + i\omega^*_{\rm F})^2 + \frac{\sigma}{\varepsilon_0}(\frac{\partial}{\partial t} - i\omega_0 + i\omega^*_{\rm F})\right]\boldsymbol{\epsilon}^*_b = \frac{1}{c^2}(\frac{\partial}{\partial t} - i\omega_0 + i\omega^*_{\rm F})^2 p^*_b\hat{\theta}^*. \tag{S17}$$

Before proceeding with Eq. (S13)–(S17), we introduce the following notation related to Fourier series. For any periodic function $f(t)$, let $f_m$ be it's $m$-th Fourier component. We define $\bar{f}$ as a column vector containing all $f_m$, thus $[\bar{f}]_m = f_m$ for $m \in \mathbb{Z}$. Additionally, we define $\bar{\bar{f}}$ as a matrix, where the $[m, n]$ entry is given by $[\bar{\bar{f}}]_{mn} = f_{m-n}$ for $m, n \in \mathbb{Z}$. In such notation, the multiplication between two functions $f(t)$ and $g(t)$ with the same period can be interpreted as the convolution of their Fourier coefficients, i.e., $\overline{fg} = \bar{\bar{f}}\bar{g} = \bar{\bar{g}}\bar{f}$ and $\overline{f^*g} = \bar{\bar{f}}^\dagger\bar{g}$, where $\dagger$ is the operation of complex-conjugate transpose.

Using the above notation, we expand Eq. (S13)–(S15) in Fourier series,

$$\bar{d}_a = 0.5\boldsymbol{\Gamma}_{\|}(\bar{\bar{E}}^\dagger_s\bar{p}_a - \bar{\bar{E}}_s\bar{p}_b + \bar{\bar{P}}_s\bar{\epsilon}_b - \bar{\bar{P}}^\dagger_s\bar{\epsilon}_a), \tag{S18}$$

$$\bar{p}_a = \boldsymbol{\Gamma}_a(\bar{\bar{D}}_s\bar{\epsilon}_a + \bar{\bar{E}}_s\bar{d}_a), \tag{S19}$$

$$\bar{p}_b = \boldsymbol{\Gamma}_b(\bar{\bar{D}}_s\bar{\epsilon}_b + \bar{\bar{E}}^\dagger_s\bar{d}_a), \tag{S20}$$

where $[\bar{\bar{E}}_{\rm s}]_{mn} = \mathbf{E}_{m-n}\cdot\hat{\theta}$ and $[\bar{\bar{P}}_{\rm s}]_{mn} = P_{m-n}$. $\boldsymbol{\Gamma}_{\|}$, $\boldsymbol{\Gamma}_a$ and $\boldsymbol{\Gamma}_b$ are diagonal matrices,

$$[\boldsymbol{\Gamma}_{\|}]_{mn} = \gamma_{\|}[m\omega_{\rm d} + \omega_{\rm F} + i\gamma_{\|}]^{-1}\delta_{mn}, \tag{S21}$$

$$[\boldsymbol{\Gamma}_a]_{mn} = \gamma_\perp[\omega_0 + m\omega_{\rm d} + \omega_{\rm F} - \omega_{ba} + i\gamma_\perp]^{-1}\delta_{mn}, \tag{S22}$$

$$[\boldsymbol{\Gamma}_b]_{mn} = \gamma_\perp[\omega_0 - m\omega_{\rm d} - \omega_{\rm F} - \omega_{ba} - i\gamma_\perp]^{-1}\delta_{mn}, \tag{S23}$$

and $\delta_{mn}$ is Kronecker delta function, $\delta_{mn} = 1$ for $m = n$, while $\delta_{mn} = 0$ for $m \neq n$. We substitute Eq. (S19)–(S20) into Eq. (S18) to cancel $\bar{p}_a$ and $\bar{p}_b$,

$$\bar{d}_a = 0.5\boldsymbol{\Gamma}_{\|}[(\bar{\bar{E}}^\dagger_s\boldsymbol{\Gamma}_a\bar{\bar{D}}_s - \bar{\bar{P}}^\dagger_s)\bar{\epsilon}_a - (\bar{\bar{E}}_s\boldsymbol{\Gamma}_b\bar{\bar{D}}_s - \bar{\bar{P}}_s)\bar{\epsilon}_b + (\bar{\bar{E}}^\dagger_s\boldsymbol{\Gamma}_a\bar{\bar{E}}_s - \bar{\bar{E}}_s\boldsymbol{\Gamma}_b\bar{\bar{E}}^\dagger_s)\bar{d}_a]. \tag{S24}$$

Eq. (S24) yields $\bar{d}_a$ as a function of $\bar{\epsilon}_a$ and $\bar{\epsilon}_b$,

$$\bar{d}_a = \boldsymbol{\chi}_a\bar{\epsilon}_a - \boldsymbol{\chi}_b\bar{\epsilon}_b, \tag{S25}$$

where

$$\boldsymbol{\chi}_a(\mathbf{r}, \boldsymbol{\omega}_{\mathbf{F}}) = 0.5[\mathbf{I} - 0.5\boldsymbol{\Gamma}_{\|}(\bar{\bar{E}}^\dagger_s\boldsymbol{\Gamma}_a\bar{\bar{E}}_s - \bar{\bar{E}}_s\boldsymbol{\Gamma}_b\bar{\bar{E}}^\dagger_s)]^{-1}\boldsymbol{\Gamma}_{\|}(\bar{\bar{E}}^\dagger_s\boldsymbol{\Gamma}_a\bar{\bar{D}}_s - \bar{\bar{P}}^\dagger_s), \tag{S26}$$

$$\boldsymbol{\chi}_b(\mathbf{r}, \boldsymbol{\omega}_{\mathbf{F}}) = 0.5[\mathbf{I} - 0.5\boldsymbol{\Gamma}_{\|}(\bar{\bar{E}}^\dagger_s\boldsymbol{\Gamma}_a\bar{\bar{E}}_s - \bar{\bar{E}}_s\boldsymbol{\Gamma}_b\bar{\bar{E}}^\dagger_s)]^{-1}\boldsymbol{\Gamma}_{\|}(\bar{\bar{E}}_s\boldsymbol{\Gamma}_b\bar{\bar{D}}_s - \bar{\bar{P}}_s). \tag{S27}$$

We substitute Eq. (S25) into Eq. (S19) and Eq. (S20) to derive $\bar{p}_{a,b}$ as functions of $\bar{\epsilon}_{a,b}$,

$$\bar{p}_a = \mathbf{\Gamma}_a[(\bar{\bar{D}}_s + \bar{\bar{E}}_s\boldsymbol{\chi}_a)\bar{\epsilon}_a - \bar{\bar{E}}_s\boldsymbol{\chi}_b\bar{\epsilon}_b], \tag{S28}$$

$$\bar{p}_b = \mathbf{\Gamma}_b[\bar{\bar{E}}_s^\dagger\boldsymbol{\chi}_a\bar{\epsilon}_a + (\bar{\bar{D}}_s - \bar{\bar{E}}_s^\dagger\boldsymbol{\chi}_b)\bar{\epsilon}_b]. \tag{S29}$$

Eq. (S28)–(S29) imply that the wave equations Eq. (S16) and Eq. (S17) are coupled through the source terms, $p_a(\boldsymbol{\epsilon}_a, \boldsymbol{\epsilon}_b)$ and $p_b(\boldsymbol{\epsilon}_a, \boldsymbol{\epsilon}_b)$ on the right-hand side. We now expand Eq. (S16) and Eq. (S17) in Fourier series,

$$-\nabla\times\nabla\times\bar{\boldsymbol{\epsilon}}_a + \frac{1}{c^2}(\varepsilon_c\boldsymbol{\omega}_a^2 + i\frac{\sigma}{\varepsilon_0}\boldsymbol{\omega}_a)\bar{\boldsymbol{\epsilon}}_a = -\frac{1}{c^2}\boldsymbol{\omega}_a^2\bar{p}_a\hat{\theta}^*, \tag{S30}$$

$$-\nabla\times\nabla\times(\bar{\boldsymbol{\epsilon}}_b)^* + \frac{1}{c^2}(\varepsilon_c{\boldsymbol{\omega}_b^\dagger}^2 + i\frac{\sigma}{\varepsilon_0}\boldsymbol{\omega}_b^\dagger)(\bar{\boldsymbol{\epsilon}}_b)^* = -\frac{1}{c^2}{\boldsymbol{\omega}_b^\dagger}^2(\bar{p}_b)^*\hat{\theta}^*, \tag{S31}$$

where $\boldsymbol{\omega}_{a,b}$ are diagonal matrices: $[\boldsymbol{\omega}_a]_{mn} = (\omega_0 + m\omega_\mathrm{d} + \omega_\mathrm{F})\delta_{mn}$ and $[\boldsymbol{\omega}_b]_{mn} = (\omega_0 - m\omega_\mathrm{d} - \omega_\mathrm{F})\delta_{mn}$. The spatial derivative operator $(-\nabla\times\nabla\times)$ acts element-wisely on each Fourier component of $\boldsymbol{\epsilon}_{a,b}$. To combine Eq. (S28)–(S31) into a group of linear equations, we must take the complex conjugate of Eq. (S31), which also flips the boundary conditions. In doing so, we derive the stability equations for the limit cycle,

$$-\nabla\times\nabla\times\begin{bmatrix}\bar{\boldsymbol{\epsilon}}_a\\ \bar{\boldsymbol{\epsilon}}_b\end{bmatrix} + \mathbf{\Omega}(\omega_\mathrm{F})\begin{bmatrix}\bar{\boldsymbol{\epsilon}}_a\\ \bar{\boldsymbol{\epsilon}}_b\end{bmatrix} = \mathbf{X}(\mathbf{r}, \omega_\mathrm{F})\begin{bmatrix}\hat{\theta}^*(\hat{\theta}\cdot\bar{\boldsymbol{\epsilon}}_a)\\ \hat{\theta}(\hat{\theta}^*\cdot\bar{\boldsymbol{\epsilon}}_b)\end{bmatrix}, \tag{S32}$$

where

$$\mathbf{\Omega} = \frac{1}{c^2}\varepsilon_c\left(\begin{bmatrix}\boldsymbol{\omega}_a & \mathbf{0}\\ \mathbf{0} & \boldsymbol{\omega}_b\end{bmatrix}^2 + i\frac{\sigma}{\varepsilon_0}\begin{bmatrix}\boldsymbol{\omega}_a & \mathbf{0}\\ \mathbf{0} & \boldsymbol{\omega}_b\end{bmatrix}\right), \tag{S33}$$

$$\mathbf{X} = -\frac{1}{c^2}\begin{bmatrix}\mathbf{\Gamma}_a\boldsymbol{\omega}_a^2 & \mathbf{0}\\ \mathbf{0} & \mathbf{\Gamma}_b\boldsymbol{\omega}_b^2\end{bmatrix}\begin{bmatrix}\bar{\bar{D}}_s + \bar{\bar{E}}_s\boldsymbol{\chi}_a & -\bar{\bar{E}}_s\boldsymbol{\chi}_b\\ \bar{\bar{E}}_s^\dagger\boldsymbol{\chi}_a & \bar{\bar{D}}_s - \bar{\bar{E}}_s^\dagger\boldsymbol{\chi}_b\end{bmatrix}. \tag{S34}$$

In one-dimensional system where $\partial_y = \partial_z = 0$ and $\hat{\theta} = \theta\hat{z}$, the perturbation of the electrical field can be simplified as $\bar{\boldsymbol{\epsilon}}_a = \bar{\epsilon}_a\theta^*\hat{z}$ and $\bar{\boldsymbol{\epsilon}}_b = \bar{\epsilon}_b\theta\hat{z}$. Thus, Eq. (S32) reduces to Eq. (14) in the main text,

$$\frac{d^2}{dx^2}\begin{bmatrix}\bar{\epsilon}_{ak}\\ \bar{\epsilon}_{bk}\end{bmatrix} + \mathbf{\Omega}(\omega_{\mathrm{F},k})\begin{bmatrix}\bar{\epsilon}_{ak}\\ \bar{\epsilon}_{bk}\end{bmatrix} = \mathbf{X}(x, \omega_{\mathrm{F},k})\begin{bmatrix}\bar{\epsilon}_{ak}\\ \bar{\epsilon}_{bk}\end{bmatrix}. \tag{S35}$$

where we have added back the index $k$.

## 2. PASSIVE PARAMETERS OF THE EP LASER

Supplementary Fig.**1** shows the passive refractive index of the EP laser cavity in the main text. The spectra in Fig.**3** and the phase portraits in Fig.**4** of the main text are recorded inside the gain cavity at $x_0 = 0.47L$.

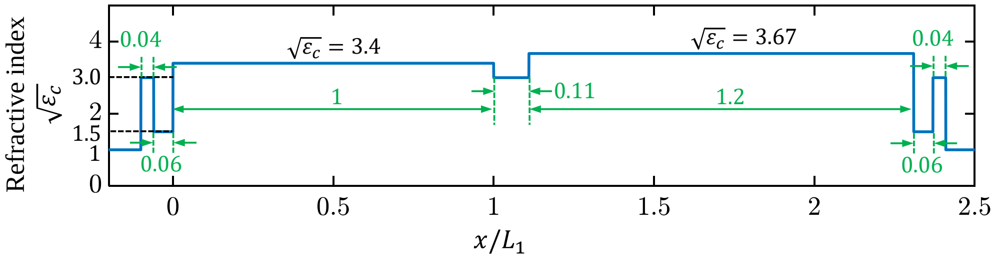


**Supplementary Fig. 1**. **The spatial profile of the refractive index in the EP laser**.

## 3. SIMULATION RESULTS FOR MORE PERIOD DOUBLINGS ABOVE $C4$

In Supplementary Fig. **2**, we present FDTD simulations of the lasing spectra for $D_\mathrm{max} > 6.0$, where solving PALT becomes impractical due to the large number of comb lines. The spectra exhibit a transition from a discrete comb structure to a continuous spectrum around $D_\mathrm{max} = 7.36$. This transition may result from either an infinite cascade of period doublings or a random crossing of the real axis by a Floquet frequency $\omega_\mathrm{F}$.

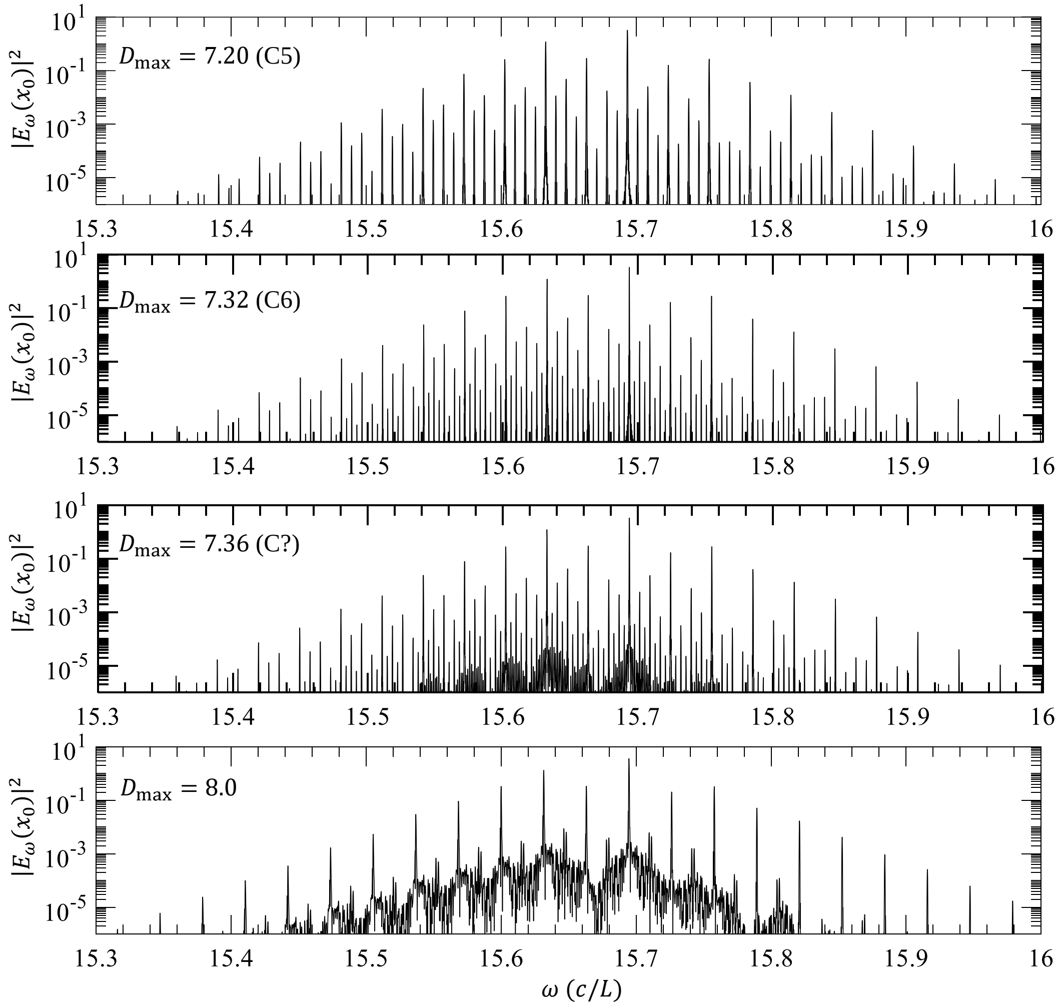


**Supplementary Fig. 2**. **Simulation results above** $C4$. Two more period doublings are observed at $D_{\mathrm{max}} = 7.20$ (first row) and $D_{\mathrm{max}} = 7.32$ (second row). At $D_{\mathrm{max}} = 7.36$ (third row), the lines are too close to be resolved. This is near the threshold between combs and continuous spectra. At $D_{\mathrm{max}} = 8.0$, the spectrum is continuous, as these results are converged for long simulation times.

[1] Gao, X., He, H., Sobolewski, S., Cerjan, A. & Hsu, C. W. Dynamic gain and frequency comb formation in exceptional-point lasers. *Nature Communications* **15**, 8618 (2024).
